# Core-level nonlinear spectroscopy triggered by stochastic X-ray pulses

**DOI:** 10.1038/s41467-019-12717-1

**Published:** 2019-10-18

**Authors:** Yves Kayser, Chris Milne, Pavle Juranić, Leonardo Sala, Joanna Czapla-Masztafiak, Rolf Follath, Matjaž Kavčič, Gregor Knopp, Jens Rehanek, Wojciech Błachucki, Mickaël G. Delcey, Marcus Lundberg, Krzysztof Tyrała, Diling Zhu, Roberto Alonso-Mori, Rafael Abela, Jacinto Sá, Jakub Szlachetko

**Affiliations:** 1 Physikalisch-Technische Bundesanstalt, Abbestr. 2-12, 10587 Berlin, Germany; 2 Paul Scherrer Institut, 5232 Villigen PSI, Switzerland; 3 Institute of Nuclear Physics Polish Academy of Sciences, 31–342 Krakow, Poland; 4 Institut Jožef Stefan, Jamova 39, 1000 Ljubljana, Slovenia; 5 Institute of Physical Chemistry, Polish Academy of Sciences, 01–224 Warsaw, Poland; 6 Department of Chemistry- Ångström, Uppsala University, 751 20 Uppsala, Sweden; 7 Linac Coherent Light Source (LCLS), SLAC National Accelerator Laboratory, Menlo Park, CA 94025 USA; 8 Present Address: Advanced Accelerator Technologies AG, 5234 Villigen PSI, Switzerland

**Keywords:** X-rays, Atomic and molecular interactions with photons

## Abstract

Stochastic processes are highly relevant in research fields as different as neuroscience, economy, ecology, chemistry, and fundamental physics. However, due to their intrinsic unpredictability, stochastic mechanisms are very challenging for any kind of investigations and practical applications. Here we report the deliberate use of stochastic X-ray pulses in two-dimensional spectroscopy to the simultaneous mapping of unoccupied and occupied electronic states of atoms in a regime where the opacity and transparency properties of matter are subject to the incident intensity and photon energy. A readily transferable matrix formalism is presented to extract the electronic states from a dataset measured with the monitored input from a stochastic excitation source. The presented formalism enables investigations of the response of the electronic structure to irradiation with intense X-ray pulses while the time structure of the incident pulses is preserved.

## Abstract (web summary)

Free electron X-ray laser pulses, generated by self-amplified spontaneous emission, are stochastic in nature. Here the authors present a reconstruction method for 2D spectroscopy while preserving the intrinsic properties of the incident pulses and apply it to a study towards X-ray intensity induced effects.

## Introduction

Stochastic processes, starting from simple Bernoulli and random walk mechanisms, mathematically describe evolving systems in different research fields like biology, chemistry, ecology or economy on the basis of random variables. In the field of X-ray physics, stochastic processes are mostly studied in the field of astrophysics when investigating the characteristics and properties of X-ray bursts from micro-quasars, black holes or the collapse of massive stars^1,2^. At the most sophisticated X-ray facilities, the X-ray free electron laser (XFEL) facilities^3^, the X-ray pulse generation is often based on a stochastic process, called self-amplified spontaneous emission (SASE). The process of spontaneous emission from undulators was proposed theoretically in the 1980s^4^ but demonstrated only 20 years later^5^. While the stochastic nature of SASE radiation is well understood and can be treated with physical models^6^, the random nature of the generated X-ray pulses leads to large uncertainties in time, space, intensity and energy and has compelled different instrumental developments for diagnostics^7,8^ while still imposing restrictions. Experimental developments were spurred as well and led to pioneering experiments regarding timing^9^ and stimulated emission^10^.

Since the advent of XFELs, X-ray spectroscopy was mainly dedicated to either studies of the unoccupied electronic states using X-ray absorption spectroscopy (XAS), or investigations of the occupied electronic states by means of X-ray emission spectroscopy (XES). Due to the mentioned stochastic nature of XFELs, XAS experiments were either realized in a scanning-based approach with a monochromatized XFEL beam^11,12^ or using a beam splitter^13,14^, a seeded XFEL beam^15,16^ and a transmissive geometry^17,18^, respectively. However, due to limited diffraction efficiencies in the beam-splitting or in the monochromatic approach only a small fraction of the incident photons is effectively used and nonlinear studies are impeded because of reduced intensities on the sample. Furthermore, the use of a monochromator extends the pulse duration significantly^19,20^. Consequently, the pulse duration of monochromatized radiation will be far above the lifetime of core level vacancies and the effectively achievable time resolution is limited. XES experiments were realized in the non-resonant regime far above the core-level ionization threshold where the stochastic nature of the incident energy distribution was less detrimental compared to XAS experiments^21–23^.

In this work a reconstruction methodology is introduced and used to demonstrate how the stochastic nature of SASE in combination with non-invasive diagnostics, in practice a transmissive spectrometer which does not affect the properties of the SASE pulses, can be used to map simultaneously the unoccupied and occupied electronic states of a scattering atom exposed to short and intense X-ray pulses. The core concept consists of using the correlation between the spectral distributions of the individual incident, non-monochromatized SASE pulses and each corresponding XES signal from the sample for a series of 10^3^–10^4^ pulses. It is shown that nonlinear X-ray processes in the vicinity of an ionization threshold can be investigated, allowing the expansion of the scope of two-dimensional spectroscopy techniques available at XFELs beyond the applications of resonant X-ray emission spectroscopy (RXES)^24^ at synchrotron radiation facilities. The discrimination of intensity-induced X-ray transparency towards opacity due to a few-eV variation of the incident photon energy will be discussed in this manuscript and attributed to the sequential ionization and excitation of atomic states with femtosecond lifetimes. The presented methodology uses SASE radiation intrinsic properties to measure the unoccupied and occupied electronic states of atoms. Effective utlilization of the most intense and, even more important, the shortest pulse durations available, is enabled and ultimately nonlinear studies on the electronic structure of a system can be realized. Hence, an attractive and possibly unique approach towards attosecond X-ray science in the vicinity of ionization thresholds is presented.

## Results

### Experimental Setup

**Fig. 1:**
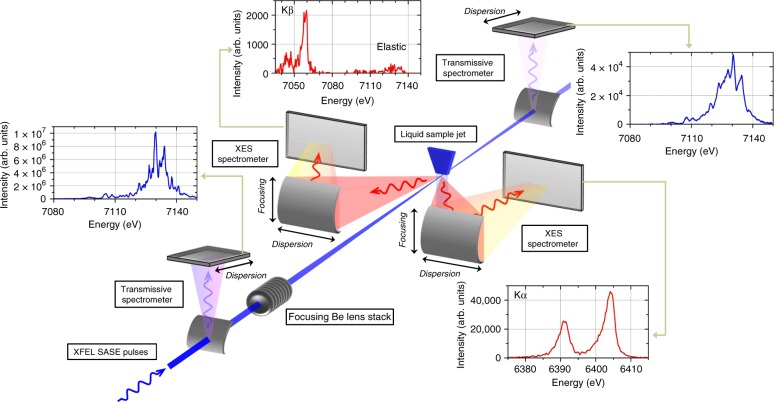
Experimental setup allowing for the use of stochastic X-ray pulses in two-dimensional spectroscopy. The spectral distribution of the incident photons was detected using a thin crystal transmissive spectrometer before they were focused by a movable Be lens stack on the liquid jet sample. The spectral distribution of the emitted X-ray photons was monitored for two X-ray emission lines with one dedicated von Hamos spectrometer, respectively. All spectrometers allow for a single-shot collection of the X-ray spectra with high energy resolution as is shown in the displayed spectra which were acquired for a single SASE pulse (insets)

The sample was an aqueous dispersion of 30 nm in diameter Fe_2_O_3_ nanoparticles which was replenished between successive XFEL pulses by means of a liquid jet setup. The spectral distribution of the individual SASE pulses was monitored using a transmissive bent crystal spectrometer with a spectral resolution of about 0.1 eV^25^ and the corresponding X-ray emission spectra of the measured Fe Kα and Kβ emission lines were recorded on a pulse-by-pulse basis using two von Hamos curved crystal X-ray spectrometers installed in a horizontal dispersion geometry^26^, as shown in Fig. 1. The introduction of dispersive X-ray spectrometers has proven to be essential since they allow for the acquisition of complete X-ray emission spectra at the full XFEL repetition rate. A Be lens stack, installed downstream of the transmissive spectrometer, was used to focus the SASE pulses on the sample. Thus, the XES source size, defined by the overlap of the incident photons with the sample volume, was small enough to preserve the intrinsic sub-eV energy resolution of the von Hamos spectrometers used. Indeed, the full potential of RXES experiments is provided when the experimental resolution is lower than the line-width broadening induced by the natural lifetime of the atomic states involved in the transition.

### Principle for the reconstruction of two-dimensional spectroscopy maps

At a hard XFEL facility each single-shot XES spectrum corresponds to the weighted sum of XES spectra produced with perfectly monochromatic incident X-ray beams of different photon energies. The relative contribution to the measured intensity is given by the number of photons in each monitored incident energy channel. The stochastic nature of the XFEL source results in intensity and mean photon energy fluctuations as well as spectral modes at different photon energies for each individual pulse. Therefore, the spectral distributions of each SASE pulse and consequently of each XES spectrum do not only vary on a pulse-to-pulse basis but are unique and cannot in principle be reproduced by a linear combination of a number of other pulses. Further, the spectral distributions of the incident SASE pulses and the XES signals are correlated through a two-dimensional matrix describing the population of the occupied and unoccupied electronic states as a function of the incident and emission energy.

### Matrix formalism

**Fig. 2:**
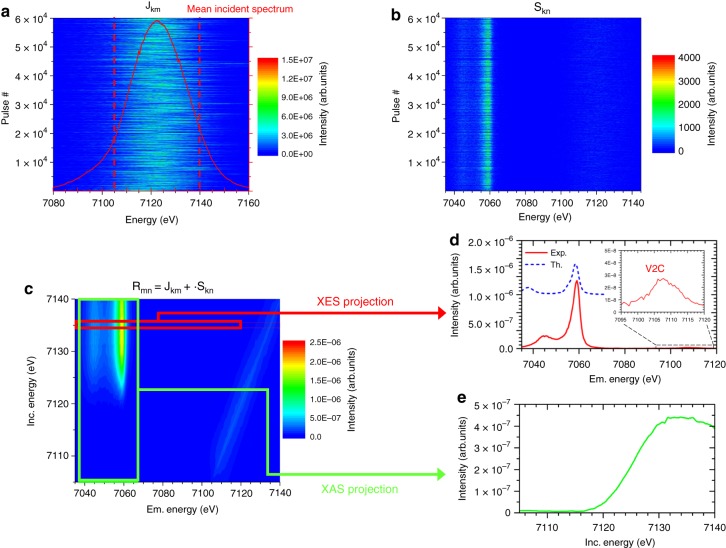
Example of the reconstruction of the two-dimensional spectroscopy map of the Kβ line. Using the spectral distribution of 60,000 incident SASE pulses **a** and of the corresponding XES produced by the broadband incident radiation **b**, the RXES map can be successfully reconstructed **c** using a matrix inversion formalism as indicated by the elastic scattering feature in the map and the XES **d**, which is compared to a multiplet calculation, and XAS spectra which can be extracted **e**. This methodology alleviates the requirement for monochromatic radiation to measure the RXES map allowing for increased efficiency of such measurements

From the synchronously recorded single-pulse maps of the incident broadband SASE pulse spectral distribution and the corresponding XES spectra, both measured in high-energy resolution, the RXES maps were reconstructed, as displayed in Fig. 2, through a matrix formalism using the intensity values of the SASE spectra and of the XES spectra as the matrix elements. One dimension of the SASE and XES matrices, labeled hereafter **J**_*km*_ and **S**_*kn*_, corresponds to the pulse number (1…*k*) and the second one to the incident and emission energy channels, respectively, where 1…*m* and 1…*n* represent the respective energy channels discriminated by the spectrometers. The RXES matrix **R**_*mn*_, describing the dependence of the spectral distribution of the emitted photons on the incident photon energy under resonance conditions, is obtained by first calculating the Moore–Penrose pseudoinverse matrix **J**_*km*_^+^^27^ and multiplying it by **S**_*kn*_, **R**_*mn*_ = **J**_*km*_^+^ ∙ **S**_*kn*_ as described in the Methods section. Thanks to the intrinsic stochasticity of the SASE pulses, the pseudoinverse matrix **J**_*km*_^+^ exists. However, due to the inherent experimental noise limiting the numerical solvability, the pseudoinverse matrix **J**_*km*_^+^ was calculated using compact single value decomposition. The RXES matrix **R**_*mn*_ was reconstructed column-wise using Tikhonov regularization^28^, with the regularization parameter being determined by means of the L-curve^29^. Furthermore it is worthwhile to note that X-ray absorption curves can also be reconstructed using the same methodology when monitoring only the X-ray fluorescence intensity emitted from the sample, **R**_*m*1_ = **J**_*km*_^+^ ∙ **S**_**k**1_.

### Reconstructed RXES maps

An example of a reconstructed RXES map **R**_*mn*_ for the Kβ line is shown in Fig. 2 together with the recorded single-shot series of SASE (**J**_*km*_) and XES (**S**_*kn*_) spectra for a total of 60,000 pulses. A qualitative validation of the reconstructed RXES maps is provided in Supplementary Fig. 1 using synchrotron radiation data for the identical sample. It has to be noted that the main absorption is reproduced at the correct position and with an identical slope than the synchrotron data while the weak pre-edge feature contained in the synchrotron RXES data is not included in the XFEL reconstructed RXES. The proneness of the reconstruction methodology to numerical errors, intrinsic to matrix inversions, and the ensuing need for numerical tools, for example regularization methods, are the likely reasons for the absence of the pre-edge feature. This issue in the reconstruction of low intensity features can be improved in future by better measurement statistics when using more efficient and possibly background-free spectrometers and detectors to improve the signal-to-noise ratios or possibly also by using a different tuning of the SASE parameters.

The standard requirement of RXES experiments for incident radiation with a narrow energy bandwidth is relaxed and throughout the experiment no scanning components are required. Hence not only dead-time is eliminated, but overcoming the necessity for a monochromator allows avoiding the expansion of the pulse duration and the rejection of the photons not fulfilling the Bragg condition. Intensity-dependent studies using the currently shortest available X-ray pulses, which are of about the same femtosecond-duration as the lifetime of the created core-level vacancy, are only enabled through the reconstruction of RXES maps. In the described approach it is mandatory that all the photons contained within an individual SASE pulse are recorded by the transmissive spectrometer in order to not neglect any contribution to the production of the XES signal in the reconstruction methodology. The RXES map itself is only reconstructed on a narrower incident energy range from 7105 eV to 7140 eV since the low photon intensity regions on the low- and high-energy side of the mean SASE spectrum are cut-out in the RXES map R_mn_ using a box filter (marked in Fig. 2). The energy of the incident X-ray pulses was broad enough to cover the resonance regime in the vicinity of the ionization threshold and allowed acquiring a complete picture of the electronic density-of-states (DOS). A series of 1200 individual XES spectra, corresponding to an acquisition time of 10 s given the 120 Hz repetition rate of the LCLS, that is on time scales which are not achievable when using standard monochromator units to scan the incident photon energy and recording the fluorescence intensity^11,30^, provides together with the spectral distribution of the corresponding SASE pulses all the information contained in the RXES map (Supplementary Fig. 2). Indeed, it is reported that between 200 and 1000 pulses are required for a proper intensity normalization of the measured fluorescence signal per measurement point in the scanned incident photon energy range^11,30^. Hence, based on a conservative estimation and considering dead time due to the necessary monochromator movement, the gain in efficiency provided by our approach is at least an order of magnitude when covering the same incident photon energy window in steps of 1 eV.

The XES spectrum and the XAS signal can be obtained from the reconstructed RXES map by projecting the average intensity from selected regions of interest onto the respective energy axis. The X-ray emission spectrum shows the Kβ line which originates from metal *3p* to *1s* electron transitions, as well as the two orders of magnitude weaker valence-to-core (V2C) line, associated to electron transitions from valence or ligand orbitals to the metallic *1s* state. The Kβ mainline is sensitive to the metal spin state and the metal-ligand covalency while the V2C region contains information about the ligand identity, electronic structure and metal-ligand bond length^31^. A calculation of the Kβ spectrum of Fe_2_O_3_ using the crystal field multiplet model (see Supplementary Note 1) gives a good overall agreement with the measured spectrum, although the splitting between Kβ_1,3_ and Kβ′ is too large due to an overestimation of the *3p*-*3d* exchange in the atomic model.

The K-edge XAS obtained from the RXES map delivers information on electron transitions from the core orbital to the conduction band and possible pre-edge features are assigned to quadrupole transitions to empty metallic *3d* states. The RXES reconstruction methodology offers a potential way to simultaneously perform XES and XAS experiments, which corresponds to a simultaneous mapping of the unoccupied and occupied electronic states, in order to follow electronic transitions with a temporal resolution solely restricted to the pulse duration.

Indeed, the most crucial aspect of the proposed methodology is that the temporal broadening related to the monochromatization of the X-ray pulses is avoided. The temporal broadening of monochromatized XFEL pulses is related to the extinction length of the X-rays in the monochromator crystal. For X-rays with a photon energy of a few keV, the extinction length amounts to few micrometers and may lead to pulse broadening of up to 20–40 fs, setting thus the physical limit for ultrafast time-resolved experiments^19,20^. A RXES map reconstructed for 5 fs-duration SASE pulses incident on the sample is shown in Supplementary Fig. 3. Regarding the short lifetimes (femtoseconds) of the excited electronic states and rearrangement processes, the ability to use the shortest available X-ray pulses is of prime importance for spectroscopy studies and currently, efforts are being made to deliver high-power and short-duration X-ray pulses at XFEL facilities^32,33^. While the impact of a monochromator on the pulse duration can be bypassed, any fundamental limits of the machine in terms of pulse duration cannot be coped with when using the reconstruction methodology.

### Incident X-ray intensity dependent studies

**Fig. 3:**
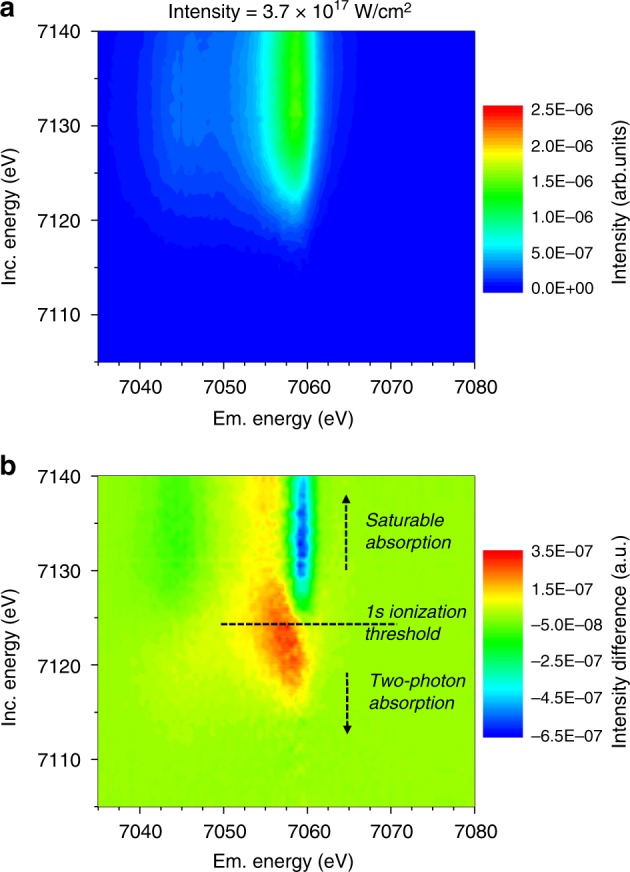
Reconstructed RXES maps for different incident intensities. The reconstructed RXES maps for the highest average X-ray intensity of the SASE pulses on the sample **a** and the difference map **b** towards the measurement at the lowest intensity, that is the RXES map from Fig. 2, **c**, are shown. In the vicinity of the core shell ionization threshold noticeable changes, with relative intensity differences of up to +17% and −32% can be observed. Notably a shift in the *1s* ionization threshold and a change in the intensity ratio between the Kβ1,3 and the Kβ′ emission lines, representative for the 3p-3d spin-coupling splitting, can be observed

As a first application of the reconstruction methodology, we opted to investigate X-ray matter interactions when increasing the incident bean intensity towards the nonlinear excitation regime in which multi-photon-in and multi-photon-out scattering processes take place and unique configurations of atomic charge states with femtosecond long lifetimes are created. The detailed information on the electronic configuration of atoms provided by RXES is necessary to acquire a complete picture of the mechanisms driving the nonlinear interaction in the vicinity of a core-level ionization threshold. It should be noted, that the reconstruction methodology does not require a priori knowledge of the physical processes involved in the X-ray matter interaction and their dependence on the total incident X-ray intensity. Only the contributions at different photon energies are considered to recover the response matrix of the system, the RXES map, which describes the response of the electronic structure as a dependence on the incident photon energy in the vicinity of an ionization threshold. Considering RXES maps reconstructed for different total incident intensities, it is shown hereafter that the reconstruction methodology enabled the simultaneous study of two non-linear X-ray processes, namely saturable absorption (SA) and two photon absorption (TPA). In the present experiment the integral intensity incident on the sample was increased from 2.2 × 10^15^ W/cm^2^ to 1.3 × 10^17^ W/cm^2^. The RXES maps obtained for the highest X-ray intensity is plotted in Fig. 3 together with the difference map with respect to the RXES map reconstructed for the lowest incident X-ray intensity displayed in Fig. 2. Energy shifts along the incident energy axis as well as along the emission energy axis are observed in the difference map.

**Fig. 4:**
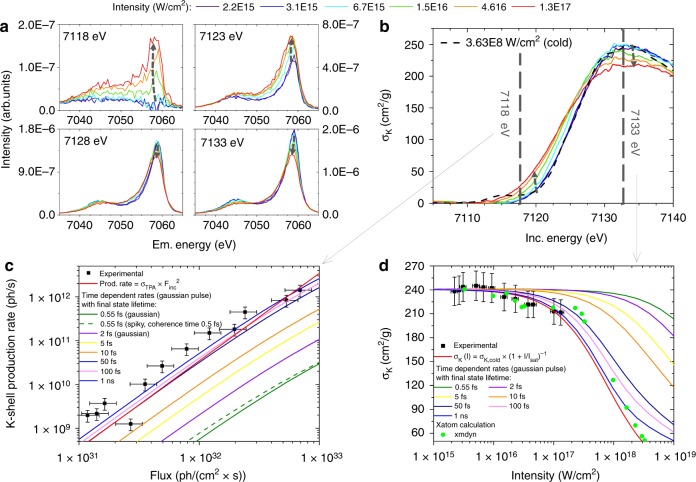
K-edge XAS and Kβ XES data showing the SA response and the increased below-edge opacity. The XES signal **a** reveals a change in the spin state of Fe_2_O_3_ with increasing incident X-ray intensity whereas the XAS behavior **b** exhibits an increased opacity below and higher transparency above the ionization threshold. Further, the TPA cross-sections for a below ionization threshold energy at 7118 eV **c** and the saturation intensity above the ionization threshold at 7133 eV **d** were derived from the recorded incident photon-energy dependent data on the K-shell production rate, respectively the photoionization cross-section. The experimental data are plotted along with the theoretical functions (red solid lines) and the rate equation calculations for both smooth Gaussian distribution (solid lines) and a spiky pulse structure (green dashed line) as well as the Xmdyn calculations on SA (green dots)

The changes in the line shape and position of the XES projections indicate an X-ray induced relative population increase of low-spin states with respect to high-spin states. Due to the interaction between the *3p* and *3d* electronic levels, the Kβ_1,3_ main line presents a sensitive probe of the Fe spin state and was used as such for characterizing the spin crossover mechanism^22,34^. The switching in the spin state results from excited states and the RXES reconstruction methodology can be used in the future as a tool for studying excitation and relaxation mechanisms and their interplay with structural changes. Indeed, the reconstructed RXES maps for different incident intensities provide more sensitive information than non-resonant XES alone as shown best by the XES projections for different photon energies plotted in Fig. 4A. It becomes evident that in the resonant regime an improved relative sensitivity towards the spin state, and a better discrimination, can be achieved by an optimized choice of the incident photon energy. This choice is only rendered possible by the complete picture provided by the RXES maps which contain all relevant fingerprints on changes induced in the occupied and unoccupied electronic states.

Simultaneously, the projection of the XAS data, displayed in Fig. 4B, shows a blueshift of the inflection point in the absorption curve by about 2 eV, changes in the absorption cross-section in the below and in the above edge region, reflected through changes in the XES intensity, and a blurring of the edge position, made obvious by a 30% increase in the peak width of the derivative of the XAS curve. The spectral differences do not reveal the contribution of resonances with possibly depopulated valence states. The photoelectric absorption cross-section σ_k_ were established using synchrotron radiation data (cold spectrum) and referencing the XAS curve extracted from the reconstructed RXES map recorded at the lowest intensity to it, assuming that the incident photon energy dependence is identical.

For incident energies below the ionization threshold, the increase in the ionization cross-sections (decreasing X-ray transparency) with increasing incident X-ray intensity can be explained through two photon absorption (TPA). The atom is ionized via a sequential two-step process during which the *1s* electron is pumped to an intermediate state followed by a second absorption process. The TPA cross-section is equal to the product of the cross-sections of each photon absorption event and the lifetime of the intermediate state. In the vicinity of an ionization threshold, the TPA mechanism may be mediated by intermediate atomic resonances^35^, as well as enhanced by higher probability for two-photon absorption via a virtual intermediate state ^36^. To determine the cross-sections for TPA, the measured XES yields for different incident X-ray intensities at an incident X-ray energy of 7118 eV were transformed to total X-ray rates as displayed in Fig. 4C. The data shows as expected a quadratic dependence and the TPA cross-section σ_TPA_ at 7118 eV was determined to be 3.4 × 10^–54^ cm^4^ s which compares reasonably well to the expected value of 1.2 × 10^–54^ cm^4^ s when considering a Z^−4^ dependence for TPA^36^.

The overall intensity decrease in the XES signal is connected to a corresponding decrease in the absorption cross-section for incident energies above the ionization threshold, displayed exemplarily for an incident energy of 7133 eV in Fig. 4D. This behavior is characteristic for SA, comparable to the few reports in the literature on nonlinear X-ray matter interaction measured in transmission experiments in the vicinity of the respective core-level ionization threshold^17,37,38^. From the measured data, the K-shell production rate was deduced and a saturation intensity I_sat_ of 7.7 × 10^17^ W/cm^2^ was determined using a simple model for SA in which it is assumed that the relaxation rate of the excited states is independent of the incident intensity^39^.

**Fig. 5:**
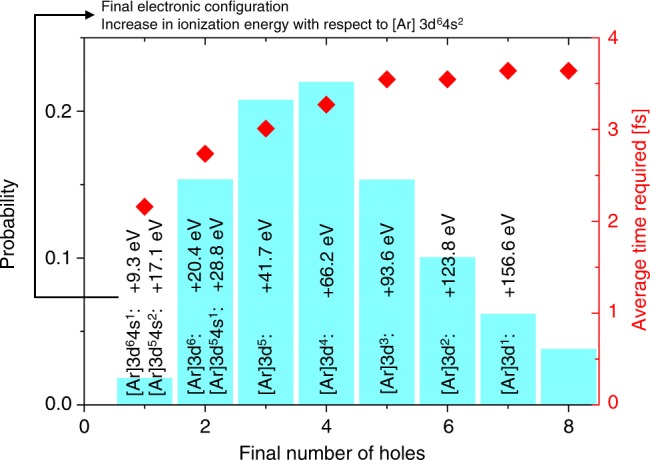
Monte Carlo simulation of the decay processes following the creation of a vacancy in the *1s* shell. Monte Carlo simulations were performed to follow the transfer of the created vacancy at the *1s* level due to radiative and Auger transitions. The simulation was performed for atomic Fe and reveals the likelihood distribution of the different valence state. The time needed for the decay cascade was also followed and was found to be considerably shorter than the duration of the SASE pulse. The shift in ionization energy for the different valence states of atomic Fe was calculated using XATOM^46^. It follows that for the higher valence states the *1s* electrons cannot be directly excited to the continuum and less likely via TPA for the incident photon energies used

The experimental observations on the intensity-dependence of the SA and the TPA were compared to calculations based on time-dependent rate equations (Supplementary Note 2) and Monte Carlo simulations (Supplementary Note 3). For SA a two-level system with a cross-section of 6.5 × 10^–20^ cm^2^^40^ was assumed for establishing the rate equations while for TPA, in agreement with the sequential excitation mechanism, a three-level system with an intermediate virtual state was used. A virtual intermediate state was considered rather than a real intermediate state involving the valence shells based on the consideration that the Kβ XES line does not provide direct evidence for the contribution of valence holes. Furthermore, the shift in the binding energy of the remaining electrons can be expected to be smaller than for the shift observed in Fig. 4B of the core-level electrons^35^. The participation of valence states in the described process cannot be excluded, but virtual intermediate states are considered to be more likely. Moreover, the basic principle underlying the mechanism for sequential TPA would be identical. For the three-level system with a virtual intermediate state a cross-section of 4.2 × 10^–21^ cm^2^^41^ for the first excitation step, a lifetime of the intermediate state of 0.11 fs^42^ and a cross-section event of 1.3 × 10^–17^ cm^2^^36^ for the second absorption were assumed. In both processes, TPA and SA, the final state is identical and has an electronic configuration has a vacancy in the *1s* shell which decays afterwards through a series of X-ray and Auger cascades with intermediate states having sub-femtosecond lifetimes. The cascade series result in charged atomic states characterized by vacancies in the inner- and outmost electronic levels and as a consequence an increased *1s* ionization threshold as compared to the ground state^43^. Monte-Carlo simulations were used to follow the transfer mechanisms of the vacancy after a *1s* ionization event and indicated, as shown in Fig. 5, that the transfer of the vacancy in the electronic configuration to the valence shell takes at most 3–4 fs after which the atom is most likely in a 3 + or 4 + valence state. As a consequence the ionization threshold for the *1s* level increases by more than 40 eV, respectively more than 60 eV for the 3 + and 4 + valence states. The calculated pronounced dependence of the ionization threshold on the charge state of the Fe atom is in agreement with existing experimental literature data on Fe^44^ and observations at XFELs on Al^43^. Due to this shift of the energy levels to higher values, multiply charged states are not accessible in the present experiment. Furthermore, solid-state effects, for example, change of atomic positions to account for charge state changes or screening effects, are considered to have only a secondary influence since the time domain probed in the experiment is limited by the pulse duration of 35 fs. Still, these effects should be confirmed experimentally.

When considering the incident photon energy distribution, a further ionization via an excitation of the remaining *1s* electron can only occur through a TPA process which is less likely by a factor of 40 because of the larger energy difference to the ionization threshold compared to the ground state configuration of the scattering atom^36,41^. In general, the change of the valence state from 1+ to N+ and the associated increase of the ionization threshold by 10 eV to 160 eV implies that further core-level ionization events can be neglected until the scattering atom returns to ground state configuration (*1s*^*2*^*2s*^*2*^*2p*^*6*^*3s*^*2*^*3p*^*6*^*3d*^*5*^ for Fe III) by electron capture processes. At the same time, the likelihood of incident X-ray photons finding an atom in a highly ionized state increases with increasing intensity.

The observed saturation at high field intensity was qualitatively reproduced using Xmdyn calculations for atomic Fe (Supplementary Note 4)^45–47^. The results of the simulation are shown in Fig. 4D. Those calculations confirmed the above predictions, namely that once a vacancy is created in the *1s* shell the ionization energy shifts due to an increase in the valence state during the transfer of the vacancy to the valence shells, as displayed in Fig. 5, and hence the atom cannot reabsorb again. It is interesting to note that the experimental data could be interpreted satisfactorily while considering the case of an isolated atom in theory. Since the incident photon energies vary in the vicinity of the Fe 1s ionization threshold, mainly the ground-state population is investigated. For this reason, post-ionization processes like electron-ion collisions^43^ or charge transfers^48^ can be disregarded as prime contributors to the observed changes. Finally, it can be concluded that the selected approach to use tabulated atomic cross-sections, thus the isolated atom approach, is in first approximation sufficient to describe the experimental data. This observation is corroborated by the relatively good agreement between the dependence of the changes on the increasing incident X-ray intensity expected in theory and observed in the experimental data.

Based on the time-dependent rate equations the time needed to return to the electronic configuration of the ground state was investigated. Information on this time-lapse is very scarce in the literature, mostly due to the lack of adequate experimental methods. This is further hindered by the complexity of the vacancy transfer processes that are associated via radiative and non-radiative decays from higher atomic levels at attosecond and femtosecond lifetimes. One experimental report indicates that the recovery to the ground-state of the electronic structure after a core-level ionization may take up to nanoseconds^49^ which is considerably longer than the XFEL pulse length and would exclude further core-level ionization events because of the increased ionization threshold. Finally, different recovery times to the ground state, varying between the *1s* core-level vacancy lifetime of 0.55 fs up to 1 ns, were assumed in the calculations. In the simplest approximation, a Gaussian envelope was used to describe the temporal structure of the incident pulses. The calculated dependences are plotted in Fig. 4c, d for TPA and SA, respectively. The same dependencies on the recovery time can be observed.

Using the shortest possible lifetime of the final state with a vacancy in the *1s* shell, both the TPA and the SA data cannot be reproduced. The stochastic distribution in the temporal domain was included by assuming a spiky-structure on the top of the Gaussian envelope for the TPA rates. The temporal incident pulse structure was generated using a coherence time of 0.5 fs in agreement with the expected LCLS machine parameters^50^. Examples of the incident pulse traces used in the simulations are shown together with the calculated dependence for the second-order coherence factor in Supplementary Note 2. Compared to the smooth Gaussian envelope in the time domain an increase in the calculated TPA rates by a factor of 1.9 can be observed but still, the experimental data is largely underestimated. Indeed, the experimental data for incident intensity dependences of TPA and SA can only be reproduced when considering a lifetime of the final state which is longer than the duration of the incident SASE pulses. The time-dependent rate equations reveal that the time needed to recover the ground state electronic configuration has to be longer than 50–100 fs. Further, the simulations show that the TPA dependence on the incident intensity can indeed be described using a relatively simple three-level atomic model which was based on the assumed sequential excitation process.

## Discussion

When exposing atoms to intense X-ray beams with photon energies varying around a core-level ionization threshold a two-fold behavior is observed in terms of increased transparency above the ionization threshold accompanied by an increase in opacity below the ionization threshold. The simultaneous revelation of this inverse behavior illustrates how spectroscopy at XFELs can contribute to investigations of intense X-ray interaction with matter. Since RXES allows for an element-sensitive disentanglement of the excitation and decay paths of the atomic states, nonlinear X-ray processes, such as sequential ionization and TPA, can be explored in more detail. For example, the changes observed around the ionization threshold were in a recent report based on core-hole model calculations attributed to the high-density excitation of *1s* electrons which in turn lead to a modification of the energy levels of the remaining, unexcited *1s* electrons^17^. The two-dimensional X-ray spectroscopy results reveal, however, two nonlinear ionization channels in the vicinity of an absorption threshold with increasing incident X-ray intensity. The SA mechanism induces a decrease in absorbance above the threshold while the TPA process is connected to higher absorption rates below it. As shown in Fig. 4, the presence of both processes in the vicinity of the ionization threshold causes the observed absorption edge blurring. In the context of TPA experiments^36,51,52^ and X-ray/optical frequency summing, the reconstruction methodology can contribute to a more accurate investigation of the X-ray intensity dependence of the intermediate virtual states on the recorded TPA yields for different incident photon energies around the ionization threshold. Considering the time scales of electronic rearrangement processes, a unique advantage of using the reconstruction methodology consists in preserving the pulse duration. Indeed, the shortest available pulses are presently on the single femtosecond time scale. Sub-femtosecond pulses, which in  have thus a simliar duration than the processes in the electronic structure following ionization events, will be available in future  and will enable original studies.

Furthermore, the efficiency of the presented reconstruction methodology makes it conceivable to record RXES maps within total acquisition times ranging from 10^–3^ s to 10 s, depending solely on XFEL facility repetition rate and detector capabilities. This opens alleys for transient ultrafast (transient) RXES experiments at XFEL facilities capable of following complete electronic rearrangement processes in real time, including laser-pump X-ray probe and X-ray pump X-ray probe experiments^53^. In particular, quadrupole transitions can be studied, provided that the low intensity features can be reconstructed in details, during catalytic processes involving transition metals to gain access to the *3d* electronic states. These states are directly involved in molecular bond formation and dissociation and thus underpin enzymatic processes respectively account for numerous catalytic systems being developed.

## Methods

### XPP instrument

The experiments were conducted at the X-ray Pump–Probe (XPP) instrument^54^ of the Linac Coherent Light Source (LCLS)^3^. The number of photons per pulse and the photon pulse energy were derived from the energy loss of the electron beam and corresponded to 1.29 × 10^12^, and 1.45 mJ. The repetition rate of the machine was 120 Hz, the average X-ray pulse duration was ~35 fs (FWHM), and the mean photon energy was varied around 7123 eV by ramping the electron beam energy up and down at a 5 Hz frequency. The manipulation of the electron beam energy created a varying incident photon energy distribution for a series of SASE pulses on a broad enough range to be of interest for RXES experiments. The mean spectrum had a FWHM of 29 eV. The photon energy bandwidth of each single XFEL pulse was about 14 eV FWHM (0.2%). The short pulse duration was achieved by running the LCLS machine in the low charge mode.

For intensity monitoring purposes, scattering signals along the beam path were surveyed with diodes and used to ensure a linear response between the diodes themselves and concerning the intensity registered by a gas monitor and by the transmission spectrometer.

### Sample

The sample was a 4% wt. aqueous dispersion of iron (III) oxide nanoparticles having an average size of 30 nm to which 5 mL polypropylene glycol anti-foaming agent was added. The sample was delivered through a sapphire jet nozzle attached to a peristaltic pump drive and head which allowed for a reliable replenishing of the sample between successive XFEL pulses. The sapphire nozzle had a width of 200 μm along the beam axis. The liquid jet delivery system was mounted on a goniometer stage and adjusted such that the XFEL pulses were incident within the laminar flow regime.

### Transmissive spectrometer

The spectral distribution of each incident SASE pulse was monitored in a non-invasive manner, with high-energy resolution and in a single-shot approach with a thin bent crystal transmissive spectrometer^25^. The spectrometer was operated in vertical reflection geometry to minimize the impact from the uneven beam profile. A Si (400) crystal with a bending radius of 50 mm and a thickness of 10 μm was used to monitor a nominal energy range from 7090 eV to 7170 eV. This energy range is broad enough to cover the full bandwidth of each XFEL pulse, accounting also for the jitter in electron energy by the Vernier mode, and is more than sufficient for the incident photon energy range in the RXES maps. The central photon energy of 7130 eV had a Bragg angle of 39.822° and the nominal photon transmission of the XFEL pulses was 77%. The part of the beam which is elastically scattered was recorded with an ORCA detector. The camera has 2048 × 2048 square pixels with a side length of 6.5 μm, and is capable of a 120 Hz frame rate. It is coupled to a 30 μm YAG:Ce screen and positioned at twice the Bragg angle concerning the XFEL pulse axis. The distance separating the crystal and the camera was 250 mm. This arrangement resulted in a nominal energy range of 52 meV per pixel along the dispersive direction, which is sufficient to resolve the single spikes in the SASE spectra when considering the extremely small angular divergence of the XFEL beam.

Downstream of the sample, at a distance of about 2 meters, an identical thin bent crystal transmissive spectrometer was operated to monitor the photons transmitted through the sample. For this spectrometer a Si (220) crystal bent to a radius of 50 mm was operated at a Bragg angle of 27°.

The energy axis of the transmissive spectrometer was calibrated in conjunction with the downstream transmission spectrometer by using the large offset monochromator of the XPP instrument^55^ for the latter spectrometer and by matching the central position and standard deviation of the mean spectra acquired with both spectrometers during the energy scan. No sample and no focusing optics were inserted in the beam path during this measurement. A Fe foil upstream of the transmissive spectrometer was used to calibrate the absolute energy axis of the spectrometers. The response function of the transmissive spectrometer was established by recording at a fixed spectrometer setting the average SASE spectra at different XFEL beam energies, normalizing the spectra to the intensity registered by an upstream gas monitor and fitting a polynomial function through the peak positions of the individual mean spectra. Moreover, dark image and background corrections were applied to each recorded image.

For each measurement at a given intensity, it was imposed that only spectra within an intensity window of 2 times the standard deviation of the recorded integral intensity were considered when reconstructing the RXES maps. In addition only those spectra were considered whose center of mass in the dispersive and the non-dispersive direction, respectively, were within a window having a width of 2 times the standard deviation of the measured individual centers of mass: in the non-dispersive direction this allowed to preserve a constant source position for the von Hamos spectrometers, whereas in the dispersive direction this allowed rejecting SASE pulses where possibly a part of the spectrum was outside the monitored detector region.

### X-ray emission spectrometer

The Fe Kα and Kβ XES signals were each monitored on a single-shot basis in high-energy resolution using two separate crystal spectrometers operated in the von Hamos geometry, allowing for a scanning-free acquisition of the XES data within a relatively larger energy band^26^. Both spectrometers used cylindrically curved crystals with a radius of curvature of 250 mm mounted in a horizontal geometry to minimize the scattering background. The dispersion axis was perpendicular to the axis of the incident pulses. The crystal dimension in the dispersive and focusing directions was 50 mm and 100 mm, respectively. For the Kα line a Ge (440) crystal was used, while for the Kβ and V2C line an InSb (444) crystal was selected. The respective Bragg angles for the Kα_1_ and Kβ_1,3_ lines were 74.47° and 69.93°. Hence the distance along the dispersive axis from the crystal center to the beam axis was 64.8 mm and 91.3 mm. In both spectrometers, a position-sensitive CSPAD-140k detector (388 × 370 pixels – dispersive and non-dispersive direction, 110 × 110 μm^2^ pixel size, 120 Hz readout rate)^56^ was installed to monitor the XES signals. The detector surface contained the dispersion axis and was normal to the axis of the incident XFEL pulses. Lead shielding was used to prevent any scattering of the XFEL pulses from air or water towards the detector. For the Kα and Kβ emission lines, this resulted in an average energy bandwidth of 0.34 eV per pixel and 0.5 eV per pixel, respectively. The expected energy resolution for the described configurations was 0.5 eV for the spectrometer monitoring the Kα emission line and 0.7 eV for the one monitoring the Kβ emission line.

To extract the XES data from the CSPAD-140k detector, a region of interest along the focusing direction was defined before projecting the data on the dispersive axis. Before the projection, a dark image correction, a background correction and a pedestal were applied and permanent and temporary pixel defects were corrected for. The energy scale of the XES spectra was calibrated using the Kα spectrum recorded at the lowest intensity together with the nominal values for Fe Kα_1_ and Kα_2_ (6403.84 eV and 6390.84 eV) whereas for the Kβ signal the envelope of the elastic signal from the aqueous sample was considered to establish an energy scale which is congruent with the transmissive spectrometer using a measurement without focusing optics in the incident beam. The stability of the XES source position, which impacts directly the XES energy axis, was confirmed by the use of two von Hamos spectrometers installed on either side of the XFEL pulse axis as shown in Fig. 1 and Supplementary Figure 4.

The recorded spectra were normalized to the identical incident intensity monitoring signal registered by an air scattering diode, just like the spectra recorded by the transmissive spectrometer. For each measurement at a given intensity, it was considered that the intensity ratio registered between the spectrometers for the Kα respectively the Kβ XES signal should be constant within a tolerance of 3 times the standard deviation of the ratio measured for the individual pulses. Following the filtering of the data from the transmission and the von Hamos spectrometers, between 23,893 and 68,653 pulses, representing on average 86% of the initially recorded pulses, were used per incident X-ray intensity for the reconstruction.

### Reconstruction of the RXES maps

At synchrotron radiation facilities RXES maps are measured using monochromatized radiation and by recording at each incident photon energy *E*_*i*_, *i* varying from 1 to *m*, an X-ray emission spectrum *S*_*i*_ composed of *n* elements. Upon normalizing each incident spectrum to the incident intensity *I*_*i*_, the RXES map can be described as a matrix **R**_*mn*_, where each row corresponds to the normalized emission spectrum: **R**_*mn*_ = **J**_*mm*_^−1^ ∙ **S**_*mn*_ with **J**_*mm*_ being a diagonal matrix containing for each incident energy *E*_*i*_ the incident photon intensity *I*_*i*_ on the diagonal element and **S**_*mn*_ being a matrix in which each row corresponds to the recorded spectrum S_i_ for the incident photon energy *E*_*i*_.

At an XFEL operated in the SASE regime, the matrix **J**_*km*_ is no longer diagonal since each incident photon pulse is described as a vector of m components with non-zero intensities at different incident energy positions nor is **J**_*km*_ square since the number of recorded pulses *k* can be different from the number of incident energy components *m*. Due to the stochastic XFEL pulse generation process, each incident energy spectrum **J**_*j*_, *j* varying from 1 to *k* and *k* being larger than m when acquiring a sufficient number of SASE pulses, is linearly independent from all other incident energy spectra. Hence the matrix has a full column rank and a left inverse can be defined as (**J**_*km*_^T^ ∙ **J**_*km*_)^−1^ ∙ **J**_*km*_^T^ which is equal to the Moore-Penrose pseudoinverse matrix **J**_*km*_^+^. The X-ray emission spectrum *S*_*j*_ corresponding to the pulse with spectrum *J*_*j*_ can be described as a linear combination of the X-ray emission spectra expected for monochromatized radiation, thus the X-ray emission spectra collected over a series of k SASE pulses corresponds to **S**_*kn*_ = **J**_*km*_ ∙ **R**_*mn*_. The RXES map is reconstructed from the measurement of the spectra of the incident SASE pulses with photon energies varying around an elemental ionization threshold and of the appertaining X-ray emission spectra using **R**_*mn*_ = **J**_*km*_^+^ ∙ **S**_*kn*_. The simplest case for the reconstruction is considered in Supplementary Fig. 5. The energy resolution is in a first approximation limited only by the energy resolution with which the spectra of the incident photons and of the emitted photons were recorded. From the qualitative comparison of the RXES maps reconstructed for the lowest incident intensity to synchrotron data, as it is done in Supplementary Fig. 1, it is inferred that the energy resolution of the reconstruction methodology is of about 1 eV on the incident energy axis and better than 1 eV on the emission energy axis.

## Supplementary information

Supplementary Material

## Data Availability

Raw data were generated at the X-ray Pump Probe instrument of the Linac Coherent Light Source. Derived data supporting the findings of the presented study are available from the corresponding authors on reasonable request.
