# Supplementary material for: Core-level nonlinear spectroscopy triggered by stochastic X-ray pulses
Source: Nat Commun. 2019 Oct 18;10:4761. doi: 10.1038/s41467-019-12717-1 (PMC6802401; doi:10.1038/s41467-019-12717-1)
Supplement: Supplementary file 1 — Supplementary Material [file 41467_2019_12717_MOESM1_ESM.pdf]

Core-level nonlinear spectroscopy triggered by stochastic X-ray pulses

Supplementary Information

Yves Kayser[1,2,*], Chris Milne[2], Pavle Juranić[2], Leonardo Sala[2], Joanna Czapla-Masztafiak[3], Rolf Follath[2], Matjaž Kavčič[4], Gregor Knopp[2], Jens Rehanek[2,‡], Wojciech Błachucki[5], Mickaël G. Delcey[6], Marcus Lundberg[6], Krzysztof Tyrala[3], Diling Zhu[7], Roberto Alonso-Mori[7], Rafael Abela[2], Jacinto Sá[5,6], Jakub Szlachetko[3,*]

[1] Physikalisch-Technische Bundesanstalt, Abbestr. 2-12, 10587 Berlin, Germany

[2] Paul Scherrer Institut, 5232 Villigen PSI, Switzerland

[3] Institute of Nuclear Physics Polish Academy of Sciences, 31-342 Krakow, Poland

[4] Institut Jožef Stefan, Jamova 39, 1000 Ljubljana, Slovenia

[5] Institute of Physical Chemistry, Polish Academy of Sciences, 01-224 Warsaw, Poland

[6] Department of Chemistry- Ångström, Uppsala University, 751 20 Uppsala, Sweden

[7] Linac Coherent Light Source (LCLS), SLAC National Accelerator Laboratory, Menlo Park, CA 94025, USA

* E-Mail: yves.kayser@ptb.de, jacinto.sa@kemi.uu.se, jakub.szlachetko@ifj.edu.pl

‡ Current address: Advanced Accelerator Technologies AG, 5234 Villigen PSI, Switzerland

Supplementary Figure 1

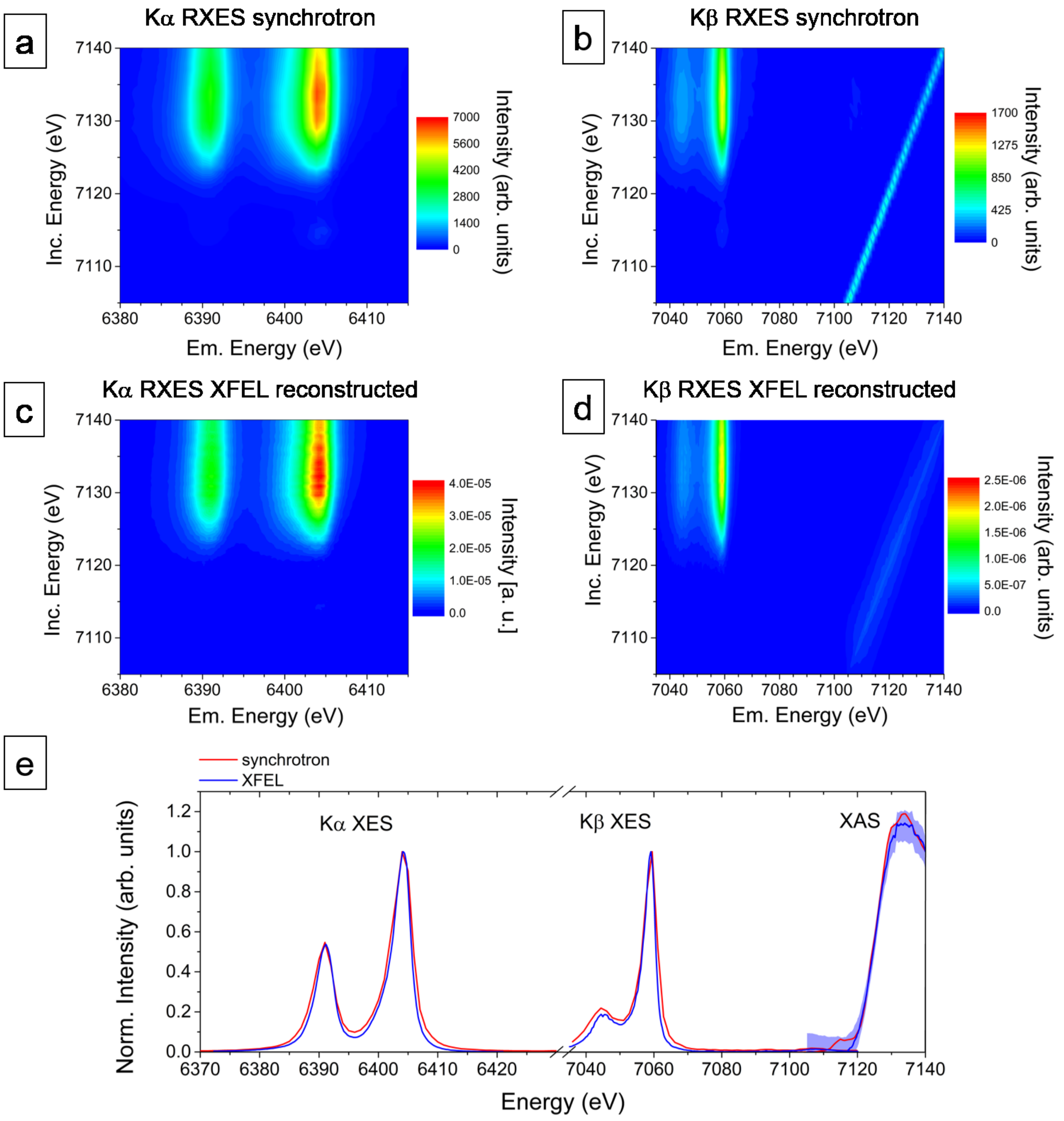

Comparison of synchrotron data to XFEL data collected at the lowest incident intensity. The RXES maps of the Kα and the Kβ emission lines measured using monochromatized synchrotron radiation (panel a, resp. panel b) are compared to the reconstructed RXES maps using non-monochromatized SASE pulses (panel c, resp. paneld). The extracted XES and XAS data is compared as well (panel e). The similarity between the RXES maps and the extracted XES and XAS data provide a qualitative validation of the reconstruction methodology. The synchrotron data was recorded at the MicroXAS beamline of the Swiss Light Source using the identical setup than for the experiment at the LCLS except for the detector and the sample concentration which was 10% wt.

during the synchrotron beamtime. A Pilatus 100K detector was used for the detection of the XES signal. The different source size, background conditions and detector pixel size, resulting in different energy resolutions for the detected XES signals make a direct comparison of the datasets tedious. Nonetheless the lineshape of the Kα and Kβ emission lines is well reproduced as well as the absorption edge and its width. This demonstrates a sufficient resolution for recording the RXES maps. However, in the XAS curve extracted from the reconstructed RXES map the pre-edge feature, which arises from *1s* to *3d* and valence band transitions, of the synchrotron data (where the total incident intensity was about ten orders more than 10 orders of magnitude lower) is not reproduced. Possible reasons for this present limitation might be the measurement precision of the transmissive and the von Hamos spectrometer (elaborate data treatment for the transmissive spectrometer to attenuate background contributions; single-shot counting statistics for the emission spectrometer). The experimental noise required and the proneness if matrix inversion algorithms to numerical errors required using numerical optimization which in turn may affect the accuracy of the final result, hence in a larger discrepancy in the pre-edge region. This could not be mediated by using a larger number of incident pulses, since the reconstructed maps are consistent with each other when varying the number of pulses for a given incident intensity (see also Supplementary Figure 2). In future the exclusive use of direct photon detecting sensors or a different tuning of the SASE beam (less spectral modes, increased jitter in energy) might help remedying the current limitations.

Supplementary Figure 2

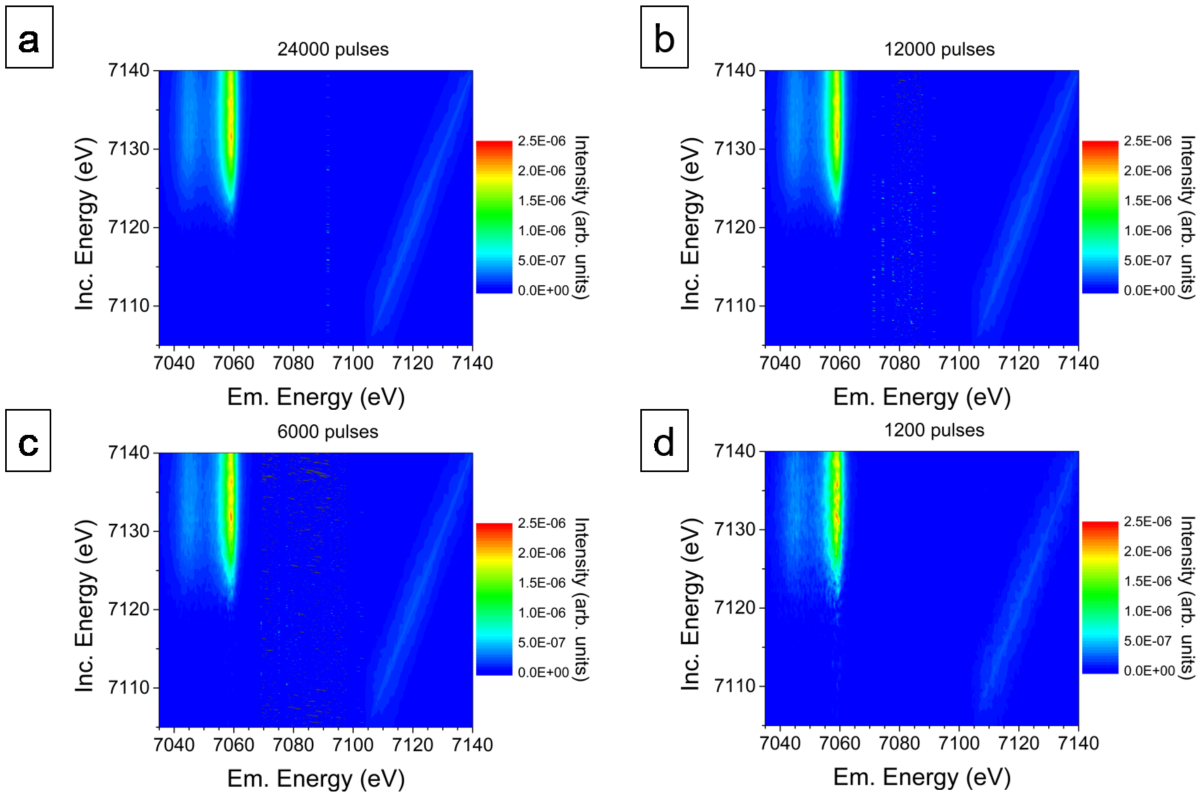

Comparison of RXES maps using pulse sequences of different lengths. The successful reconstruction of the RXES map for the Kβ lines is shown for different number of incident SASE pulses. The first 24000 (panel a), 12000 (panel b), 6000 (panel c), respectively 1200 pulses (panel d) of the single-shot spectra shown in Figure 2 (panels a and b) were used for the reconstruction of the RXES map. Given the 120 Hz repetition rate of the LCLS this shows that RXES maps can be acquired in time frames as short as 10 s. Note, that this bandwidth cannot be covered in such a short time when RXES measurements are realized using a monochromator to scan the incident energy. In this view the presented reconstruction methodology enables in future ultrafast time-resolved RXES experiments at XFELs in conjunction with pump-probe schemes.

Supplementary Figure 3

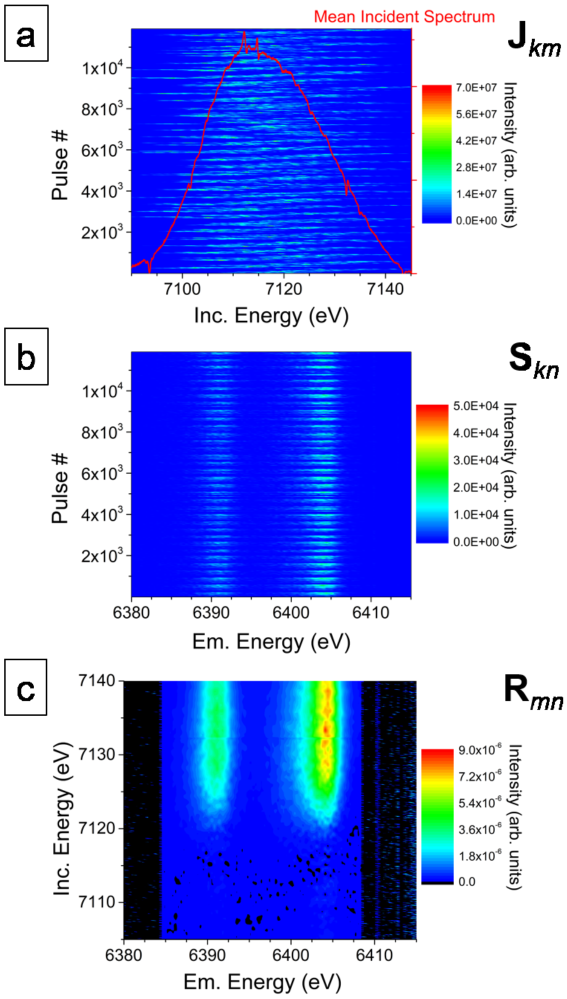

Demonstration of the reconstruction methodology using femtosecond-duration SASE pulses. The reconstruction of a two-dimensional spectroscopy map of the Fe Kα line using XFEL pulses with a duration of approximately 5 fs while avoiding any temporal broadening from monochromatization or seeding is shown. The incident SASE (panel a) and emitted XES spectra (panel b) were recorded synchronously on a pulse-to-pulse basis and are displayed in the top and middle panels, respectively. The reconstructed RXES map (panel c) is shown in the bottom panel. Regions where the reconstruction methodology presented numerical errors (intensity spikes) are blanked out. This example demonstrates that the reconstruction methodology provides a possibly unique approach towards studying X-ray interaction with matter in the vicinity of a core ionization threshold while using X-ray pulses with a similar duration than the excited electronic states and rearrangement processes.

Supplementary Figure 4

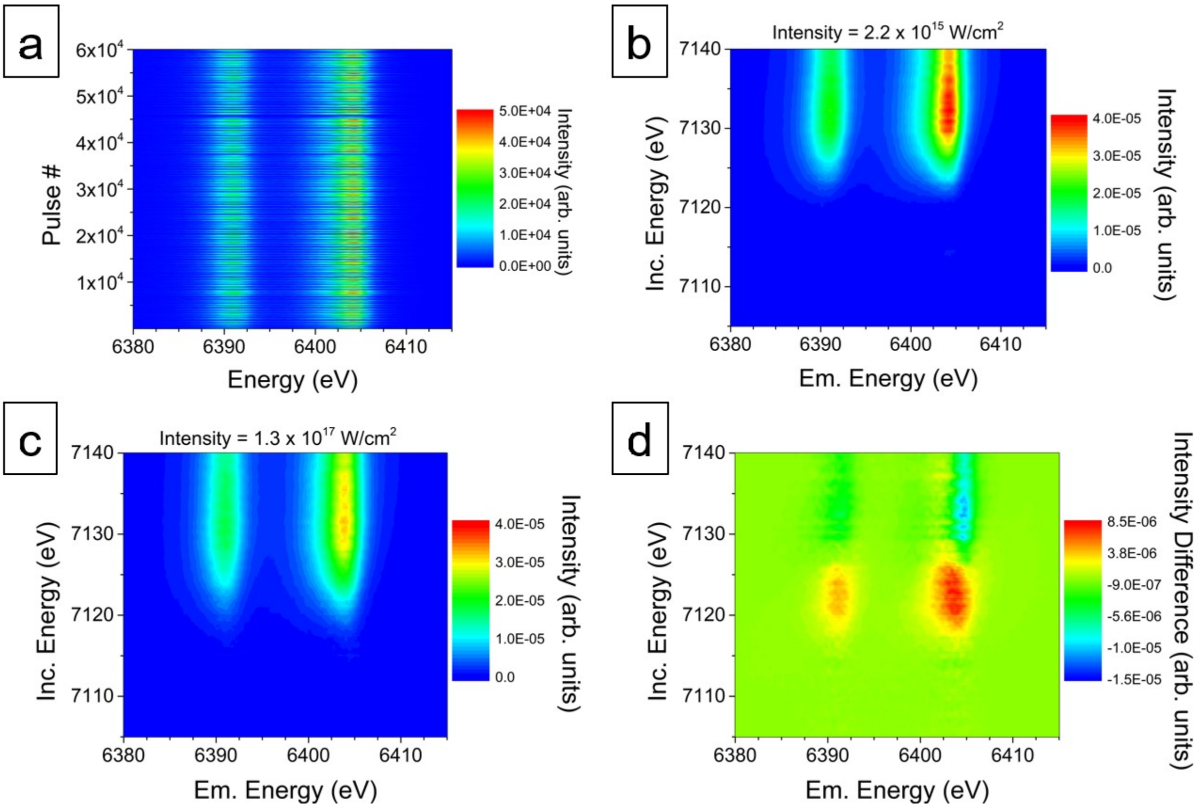

Reconstruction of Fe_2O_3 Kα RXES maps measured at the lowest and highest incident intensity. The displayed examples correspond to the measurements shown for the Kβ line in Figures 2 (panel c) and 3 (panel a). The emission spectra (panel a) measured with the SASE radiation at the lowest incident intensity (Figure 2, panel a) is displayed in the top left panel. The difference between the RXES map reconstructed for the lowest intensity (panel b) and the highest intensity (panel c) is represented in panel c. Like for the Kβ line a shift in the XAS inflection point position and a change in the XES spectral shape can be observed. As the shift is observed in the same energy direction and the spectrometers were installed on opposite sides with respect to the incident SASE pulses, this indicates a stable energy calibration throughout the experiment.

Supplementary Figure 5

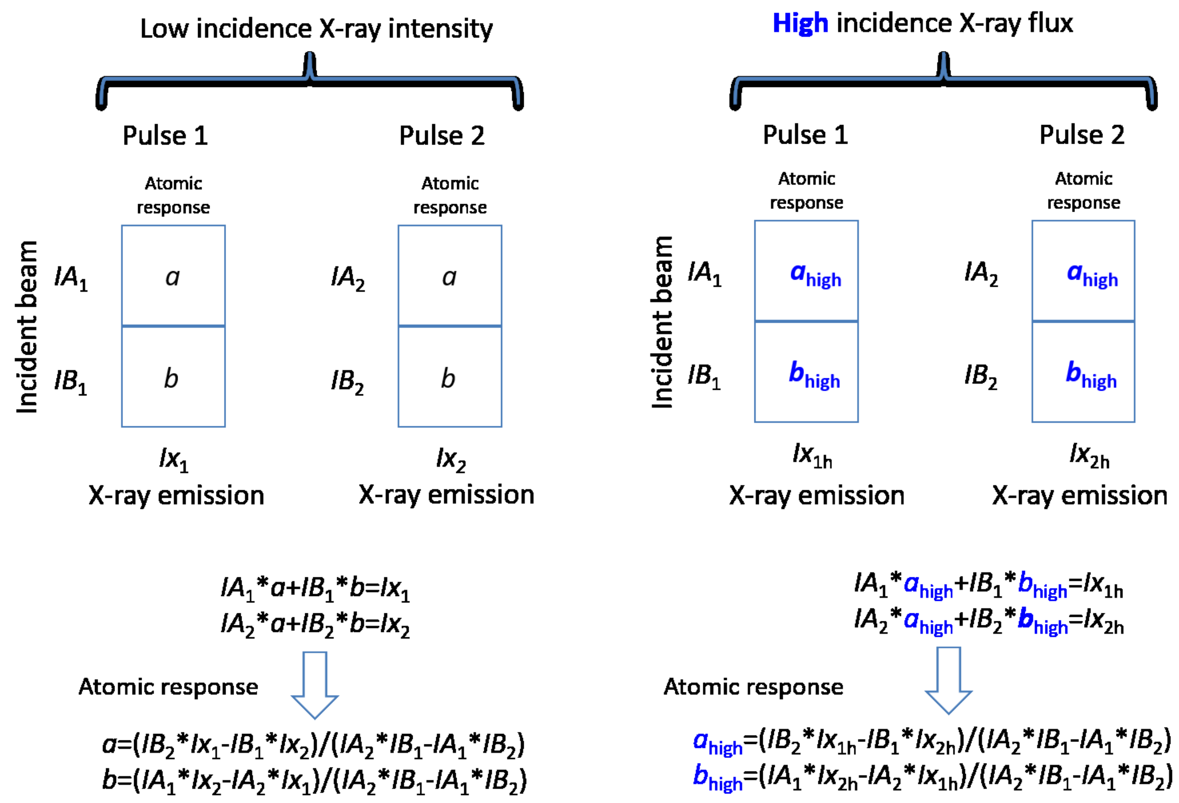

Illustration of the reconstruction methodology considering 2 incident and 1 emission energy. Basics of the mathematical reconstruction procedure on the simplest example of two different incident energies, two pulses and one emission energy (k=2, m=2, n=1):

$$\begin{bmatrix} Ix_1 \\ Ix_2 \end{bmatrix} = \mathbf{S}_{2,1} = \mathbf{J}_{2,2} \cdot \mathbf{R}_{2,1} = \begin{bmatrix} IA_1 & IB_1 \\ IA_2 & IB_2 \end{bmatrix} \cdot \begin{bmatrix} a \\ b \end{bmatrix}$$

In the experiment for each pulse Ix_g and IA_g and IB_g with g=1,2 are measured for the emission energy *Ex* and the incident energies *EA* and *EB*. Note that here only the number of photons within a given incident energy bin are considered as the intensity elements. The aim of the reconstruction methodology is to retrieve the response of the system (*a*, *b*), which corresponds to the RXES map. Since two incident energies are considered, at least two pulses need to be considered, marked by the indexes 1 and 2, to solve the system.

At the same time it is worthwhile to stress that the mathematical procedure remains fully valid when applying higher total incidence X-ray intensities in units of W/cm^2 (equivalently the total or integral number of photons per unit time and per unit area) leading to possibly to a different intensity response

of the atomic system. Indeed, the total incident intensity *I1* and *I2* is not required to be known. The nonlinear response is evaluated by comparing a and a_{high}, respectively b and b_{high} or in other words changes in the response of the system under study for different total incident intensities.

Supplementary Note 1: Crystal-field multiplet calculations

Calculations of the Kβ spectrum of Fe_2O_3 were performed using the crystal field multiplet model through the CTM4XAS interface [1]. A ligand-field splitting of 1.45 eV was used and the d-d interactions were reduced to 70% of their original values [2]. A Gaussian broadening of 2.0 eV was applied to all transitions. Different Lorentzian broadenings were used for the $K\beta_{1,3}$ (0.8 eV FWHM) and Kβ' (2.0 eV FWHM) to account for state-dependent lifetimes [3].

Supplementary Note 2: Time-dependent rate equations

The rate equations for two-photon absorption (TPA) at an incident photon energy below the ionization threshold as well as for one photon absorption (OPA) at energies above the ionization threshold were employed to determine the change in the initial and final state populations during the course of a femtosecond duration X-ray pulse. The processes of TPA and OPA in two- and three-level systems are sketched in Supplementary Figure 6.

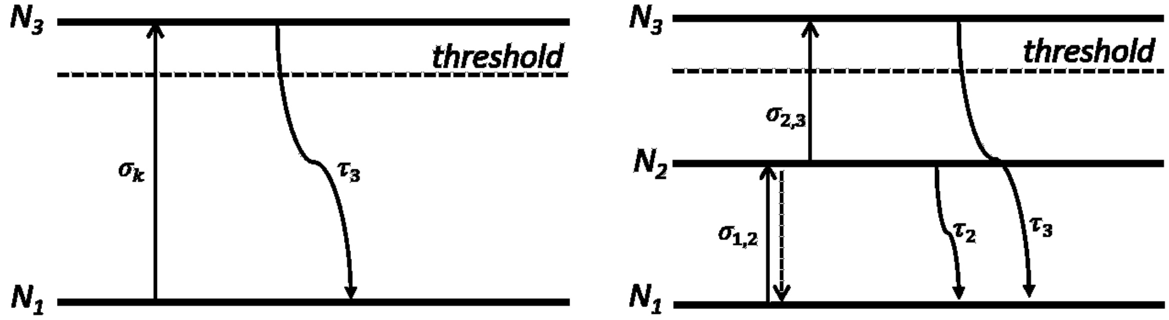

Supplementary Figure 6 **Scheme of the one photon and two photon absorption processes.** In OPA (panel a) the ionization from the ground state N_1 to the excited state N_3 is caused by a photon with an energy above the ionization threshold and the excited atom returns to the ground state after a lifetime τ_3. In the sequential TPA process (panel b), the ionization is only possible via an intermediate, virtual state N_2 from which the electron might be excited by a second photo-absorption process to the N_3 state. The return to the ground state can occur from the intermediate state (lifetime τ_2) or from the excited state (lifetime τ_3).

For the OPA process, the equations describe the depopulation of the initial states of the atoms N_1, as well as the decays from the excited states N_3 with lifetime t_3:

$$\frac{dN_1}{dt} = -I(t) * \sigma_k * N_1(t) + \frac{N_3}{\tau_3}$$

$$\frac{dN_3}{dt} = I(t) * \sigma_k * N_1(t) - \frac{N_3}{\tau_3}$$

In case of TPA, we assumed a sequential model of the process with an intermediate virtual state. The equations describe the population of initial- (N_1), intermediate (N_2) and final (N_3) states with lifetimes τ_2 and τ_3 for the intermediate and excited states respectively:

$$\frac{dN_1}{dt} = -I(t) * \sigma_{1,2} * (N_1(t) - N_2(t)) + \frac{N_2}{\tau_2} + \frac{N_3}{\tau_3}$$

$$\frac{dN_2}{dt} = I(t) * \left[\sigma_{1,2} * (N_1\,(t) - N_2(t)) - \sigma_{2,3} * N_2(t)\right] - \frac{N_2}{\tau_2}$$

$$\frac{dN_3}{dt} = I(t) * \sigma_{2,3} * N_2(t) - \frac{N_3}{\tau_3}$$

The following cross-sections and lifetimes values were used in the calculations:

- $\sigma_{1,2} = 4.2 * 10^{-21}$ cm^2 at an energy of 7118 eV. This value is obtained by using $\sigma_{1,2}$ value of atomic Fe [4] and rescaling it by a factor of 1.4 based on the cross-section ratio between Fe and Fe_2O_3 for photon energies above the ionization threshold.
- $\sigma_{2,3} = 1.3 * 10^{-17}$ cm^2 – the cross-section value for Cu from [5].
- $\sigma_{OPA} = 6.5 * 10^{-20}$ cm^2 – the cross - section value for Fe_2O_3 taken from NIST XCOM database for an incident photon energy of 7133 eV.
- $\tau_2 = 1.1 * 10^{-16}$ s – calculated from the equation describing scattering time [6]: $\tau_2 = \frac{\hbar}{\sqrt{(E_i - E_1)^2 + \Gamma_i{}^2}}$.
- $\tau_3 = 5.5 * 10^{-16}$ s – tabulated lifetime for the *1s* core state [7].

The same calculations were also performed for ground state recovery times of 2, 5, 10, 50, 100 fs and 1 ns.

For the time-dependent simulations, the intensity of the incident X-ray pulse was assumed to have a Gaussian distribution with a FWHM of 35 fs. To account for the stochastic nature of the incident X-ray pulses, calculations were performed assuming a spiky structure on the top of the Gaussian envelope for 100 randomly generated pulse shapes. The pulse structures used in the calculations are plotted in Supplementary Figure 7.

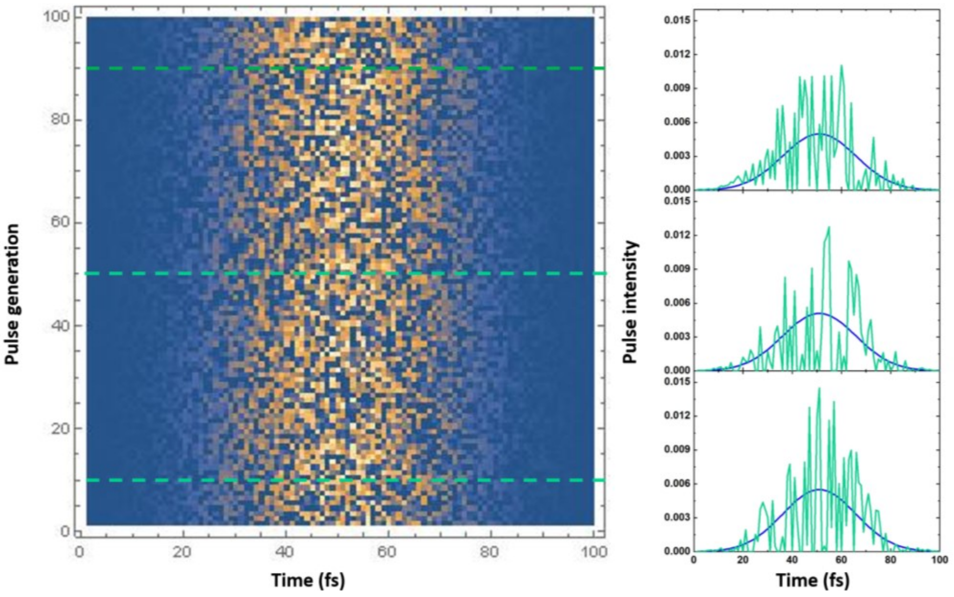

Supplementary Figure 7 **Density plot of the randomly generated X-ray pulses used for time-dependent calculations.** On the right side, examples of the three temporal distributions are plotted. The blue line corresponds to the Gaussian intensity distribution with FWHM of 35 fs.

For the temporal spiky pulse structure, the degree of second order of coherence, $g^{(2)}$ was calculated with the following equation:

$$g^{(2)} = \frac{\langle I_{(t)} I_{(t+\tau)} \rangle}{\langle I_{(t)} \rangle^2}$$

and the results of the calculation are plotted on the Supplementary Figure 8.

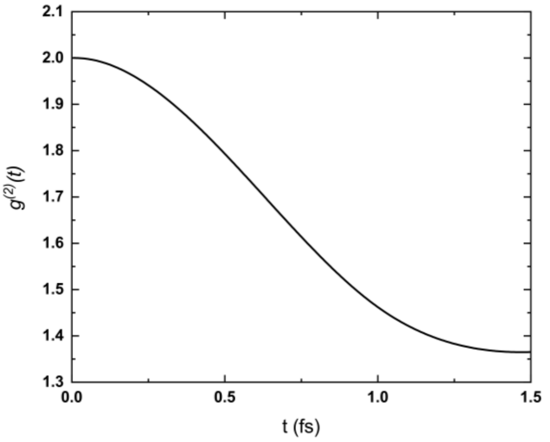

Supplementary Figure 8 **Degree of second order coherence for randomly generated incident X-ray pulses.** From $g^{(2)}$ the coherence time of simulated pulse was estimated to be 0.5 fs.

Supplementary Note 3: Monte Carlo simulation of the vacancy transfer

Monte Carlo simulations of all possible fluorescence and Auger decay cascades were realized starting from one electron vacancy in the K-shell of a metallic Fe atom with the initial electronic configuration $1s^2 2s^2 2p^6 3s^2 3p^6 3d^6 4s^2$. The creation of an unoccupied state in the $1s^2$ level is the most likely event in the experiment realized. In the simulation the type of decay, fluorescence or Auger, is first randomly selected using the corresponding Auger yield [8]. In case of a fluorescence decay, the vacancy in the electronic configuration is removed and another one is created on an energy level drawn using X-ray transition probabilities [9] as weighting factors. In case of an Auger decay, the vacancy is removed and two are created at energy levels taken from an Auger decay path chosen randomly among the allowed ones. At every electron vacancy removal and creation a decay schedule is updated using reported lifetime broadenings [8]. The decay schedule is used to process the transfer of the vacancies decays in the correct time sequence and allows determining the temporal evolution of energy levels' occupancies. Once an electron vacancy has been processed and the decay schedule has been updated, the simulation treats the next scheduled electron vacancy decay in the described way and the algorithm continues iteratively. If the algorithm encounters an electron vacancy which occupies a zero-Auger yield energy level or if higher energy levels are empty of electrons, the electron vacancy is left at its position and the next scheduled one is processed. The simulation stops when the decay schedule contains no more electron vacancies which can be processed.

Supplementary Note 4: Xraypac simulations

The field-dependent X-ray-induced dynamics simulations were performed using the Xmdyn module in Xraypac [10-14]. As Xmdyn simulations can only start from the standard atomic configuration, atomic iron was used as the model system. The incident energy was chosen 2 eV above the calculated ionization edge for iron. This is below the ionization energy for Fe+ and therefore, once ionized, the system cannot have further *1s*-ionisation. The simulations were performed at different incident intensities and between 200 and 20000 independent runs were computed for each intensity in order to obtain proper statistics. More runs are required to obtain converged results for the lowest intensities since excitations events are less likely. A constant intensity shift and cross-section scaling was applied in order to align the calculated results to the experimental data.

The Xmdyn results for the iron atom agree very well with a simple analytical model based only on the assumption that after a first ionization, regardless of the relaxation path, no further *1s*-ionisation is possible. This model depends only on the 1s and total absorption cross-sections and can therefore be applied to Fe III using the absorption cross-sections and the transition energies computed with the XATOM program [14]. The results for this model of Fe_2O_3 are also qualitatively consistent with the experimental results.

Supplementary Bibliography

1. Stavitski, E. & De Groot F. M. F., The CTM4XAS program for EELS and XAS spectral shape analysis of transition metal L edges. *Micron* **41**, 687-694 (2010).
2. Kuiper, P., G., S. B., Rudolf, P., Tjeng, L. H. & Chen, C. T., X-ray magnetic dichroism of antiferromagnet Fe2O3: The orientation of magnetic moments observed by Fe 2p x-ray absorption spectroscopy. *Phys. Rev. Lett.* **70**, 1549 (1993).
3. Glatzel, P., Bergmann, U., de Groot, F. M. F. & Cramer, S. P., Influence of the core hole on Kβ emission following photoionization or orbital electron capture: A comparison using MnO and 55Fe2O3. *Phys. Rev. B* **045109**, 64 (2001).
4. Sánchez, H. J., Valentinuzzi, M. C. & Pérez, C., X-ray resonant Raman scattering cross sections of Mn, Fe, Cu and Zn. *J. Phys. B At. Mol. Opt. Phys.* **39**, 4317 (2006).
5. Szlachetko, J. *et al.*, Establishing nonlinearity thresholds with ultraintense X-ray pulses. *Sci. Rep.* **6**, 33292 (2016).
6. Gel'mukhanov, F., Sałek, P., Privalov, T. & Agren, H., Duration of X-Ray Raman Scattering. *Phys. Rev A* **59**, 380 (1999).
7. Campbell, J. L. & Papp, T., Widths of the Atomic K-N7 Levels. *Atom. Data Nucl. Data* **77**, 1-56 (2001).
8. Schoonjans, T. *et al.*, The xraylib library for X-ray–matter interactions. Recent developments. *Spectrochim. Acta, Part B* **66**, 776 – 784 (2011).
9. Thompson, A., "X-ray Properties of the Elements" in X-ray Data Booklet (Lawrence Berkeley Laboratory, Berkeley, USA, ed. 3, 2009), pp. 9-64.
10. Jurek, Z., Santra, R., Son, S.-K. & Ziaja, B., XRAYPAC – a software package for modeling x-ray-induced dynamics of matter, v. 1.01, CFEL, DESY (2018).

11. Jurek, Z., Son S.-K. & Ziaja, B. & Santra, R., XMDYN and XATOM: versatile simulation tools for quantitative modeling XFEL-induced dynamics of matter. *J. Appl. Crystallogr.* **49**, 1048 (2016).

12. Son, S.-K., Young, L. &. Santra, R., Impact of hollow-atom formation on coherent x-ray scattering at high intensity. *Phys. Rev. A* **83**, 033402 (2011).

13. Jurek, Z., Faigel, G. & Tegze, M., Dynamics in a cluster under the influence of intense femtosecond hard X-ray pulses. *Eur. Phys. J. D* **29**, 217 (2004).

14. Murphy, B. F. *et al.*, Femtosecond X-ray-induced explosion of C60 at extreme intensity. *Nat. Commun.* **5**, 4281 (2014).
